# Veterinary Medical Society of Ontario College

**Published:** 1890-05

**Authors:** 


*Secretary.*

---

*Veterinary Medical Society of the Ontario College.*—This society, after a most successful Winter's work, is holding its closing meetings for the session 1889-90. A well-attended meeting was held last evening (March 24th) in the large lecture hall of the college, at which some very excellent papers were read, the first being upon "Diarrhœa of Nurslings," by Mr. F. W. Menhenitt. Mr. J. H. Ullrich's paper was read next, upon "Omphalitis," a remarkably good and practical essay. He accepts and advocates the theory of Bollinger, viz.: that the arthritis is due to the previous condition of the urachus, and a cure is to be sought by the immediate treatment of that condition. The umbilical vein is to be injected with a weak corrosive sublimate solution, the animal, of course, being turned upon his back. If the urachus itself is to be injected, the essayist advises not the corrosive sublimate, but a boracic acid solution. Appropriate tonics are also advised.

A lively discussion ensued, some of the members speaking of ligature as a means of treatment. Mr. Skirrow, in his remarks, condemned the use of the ligature, as tending to fatal results, but spoke well of an injection of a strong solution of tannic acid. Cases so treated had made good recovery.

Mr. L. A. Wright's paper, on the "Workings of Heredity," was listened to with marked attention. The points discussed, and the conclusions arrived at, cannot be dealt with in a short report like the present, but were ably and convincingly put forward. The spirited discussion which ensued brought out the varying opinions of the members. Mr. Foiles held that there is in some horses an hereditary tendency to lymphangitis, with which theory the essayist could not agree. Mr. C. Pink read an essay on "Lymphangitis," Mr. G. Kerr one on "Sprains," and Mr. C. A. Keene an admirable one on "Shoeing." Communications from as many members, on "Amputations," "Tumor on the Brain," "Lead Poisoning." The president of the society (Professor Smith) was in the chair. Much of the success of this society during the past season has been due to the labors of its popular and efficient secretary, Mr. W. J. Broaddus. The staff of instructors of the Ontario Veterinary College has been enlarged by the addition of C. Sweetapple, V.S., as lecturer on obstetrics. His course of instruction in this important department has been much appreciated by the students.

The new building of the college has been in use since New Year's. Its excellent arrangements, especially in regard to its lecture and class rooms, have given great satisfaction to teachers and students.

The written examinations are now being held in all the classes. The oral for the Juniors took place on the evening of March 22d. A large number went up to examination, and a high standard of excellency was generally observable.

